# Blood pressure change does not associate with Center of Pressure movement after postural transition in geriatric outpatients

**DOI:** 10.1186/s12877-017-0702-2

**Published:** 2018-01-15

**Authors:** Sjoerd T. Timmermans, Esmee M. Reijnierse, Jantsje H. Pasma, Marijke C. Trappenburg, Gerard J. Blauw, Andrea B. Maier, Carel G. M. Meskers

**Affiliations:** 1 Department of Rehabilitation Medicine, VU University Medical Center, PO Box 7057, 1007 MB Amsterdam, The Netherlands; 2 Department of Medicine and Aged Care, Royal Melbourne Hospital, University of Melbourne, Melbourne, Australia; 3 Department of Biomechanical Engineering, Delft University of Technology, Delft, The Netherlands; 4 Department of Internal Medicine, Section of Gerontology and Geriatrics, VU University Medical Center, Amsterdam, The Netherlands; 5 Department of Internal Medicine, Amstelland Hospital, Amstelveen, The Netherlands; 6 Department of Gerontology and Geriatrics, Leiden University Medical Centre, Leiden, The Netherlands; 7 Department of Internal Medicine, Haaglanden Medical Center Bronovo, The Hague, The Netherlands; 8 Department of Human Movement Sciences, Amsterdam Movement Sciences, Amsterdam, Vrije Universiteit, Amsterdam, The Netherlands

**Keywords:** Orthostatic hypotension, Center of pressure, Standing balance, Blood pressure, Aged

## Abstract

**Background:**

Orthostatic hypotension (OH), a blood pressure drop after postural change, is associated with impaired standing balance and falls in older adults. This study aimed to assess the association between blood pressure (BP) and a measure of quality of standing balance, i.e. Center of Pressure (CoP) movement, after postural change from supine to standing position in geriatric outpatients, and to compare CoP movement between patients with and without OH.

**Methods:**

In a random subgroup of 75 consecutive patients who were referred to a geriatric outpatient clinic, intermittent BP measurements were obtained simultaneously with CoP measurements in mediolateral and anterior-posterior direction directly after postural change during 3 min of quiet stance with eyes open on a force plate. Additional measurements of continuous BP were available in *n* = 38 patients. Associations between BP change during postural change and CoP movement were analyzed using Spearman correlation. Mann-Whitney-U tests were used to compare CoP movement between patients with OH and without OH, in which OH was defined as a BP drop exceeding 20 mmHg of systolic BP (SBP) and/or 10 mmHg of diastolic BP (DBP) within 3 min after postural change.

**Results:**

OH measured intermittently was found in 8 out of 75 (11%) and OH measured continuously in 22 out of 38 patients (57.9%). BP change did not associate with CoP movement. CoP movement did not differ significantly between patients with and without OH.

**Conclusions:**

Results do not underpin the added value of CoP movement measurements in diagnosing OH in a clinical setting. Neither could we identify the role of CoP measurements in the understanding of the relation between OH and impaired standing balance.

**Electronic supplementary material:**

The online version of this article (10.1186/s12877-017-0702-2) contains supplementary material, which is available to authorized users.

## Background

Impaired standing balance is commonly present in older adults [1–3] and is associated with falls, hospitalization, impaired quality of life, extensive morbidity and mortality [2, 4–6]. Standing balance is regulated through the interaction of the sensory, motor and nervous systems [5, 7]. These key systems deteriorate with advanced age, diseases and medication use [8, 9]. It is important to distinguish the underlying causes of impaired standing balance for the development of targeted interventions to improve standing balance and finally prevent falls [7]. Orthostatic hypotension (OH), and especially initial OH (iOH), is significantly associated with impaired standing balance in older adults [10, 11] and therefore with falls [6, 12–14]. Orthostatic hypotension (OH), a drop in systolic BP (SBP) of at least 20 mmHg and/or a drop in diastolic BP (DBP) of 10 mmHg or more within 3 min of standing position, is associated with impaired standing balance and falls in older adults [15]. iOH is defined as a transient decrease in BP within 15 s after postural change with a decline in SBP of at least 40 mmHg and/or 20 mmHg in DBP [16]. OH is the most prevalent blood pressure (BP) regulation disorder in older adults after hypertension [15], with a prevalence of five to 30% in community-dwelling older adults and geriatric outpatients when BP was measured intermittently [17–20] and 57–94% in geriatric outpatients when BP was measured continuously [14, 20].

The few studies so far that investigated the relation between BP change and standing balance [3, 11, 20, 21] showed increased Center of Mass (CoM) movement during stance in both community-dwelling older adults and Parkinson patients with OH compared to patients without OH [3, 21]. In geriatric outpatients, OH was found to be associated with the ability to maintain standing balance during semi-tandem stance with eyes closed. In addition, OH was associated with an increase in self-reported impaired standing balance [20]. Another way to measure standing balance is by the quality of standing balance. Center of Pressure (CoP) movement portrays the quality of balance by measuring the movement of the application point of corrective forces needed to keep the body balanced [22, 23]. Previous studies addressed measures of standing balance i.e., CoM movement, maintenance of balance during semi-tandem stance and self-reported balance in relation to orthostatic BP change [3, 11, 20, 21]. CoP movement adds to that as a measure of the quality of standing balance by addressing the movement of the application point of corrective forces needed to keep the body upright [22, 23]. Additionally, we measured CoP and BP simultaneously after postural change as opposed to other studies that measured standing balance during quiet stance and BP change non-simultaneously. Literature on the relation between OH, BP change and the quality of standing balance and the relation between BP change and the quality of standing balance directly after standing up in geriatric outpatients is currently lacking.

The aim of this study was to assess the association between BP change and the quality of standing balance, directly after postural change in a clinically relevant population of geriatric outpatients. It is hypothesized that larger BP change after postural change is associated with higher CoP movement as an indication of impaired quality of standing balance. Furthermore, it is hypothesized that patients with OH differ in quality of standing balance, hence, exhibit increased CoP movement after postural change, compared with patients without OH.

## Methods

### Study design

**Fig. 1:**
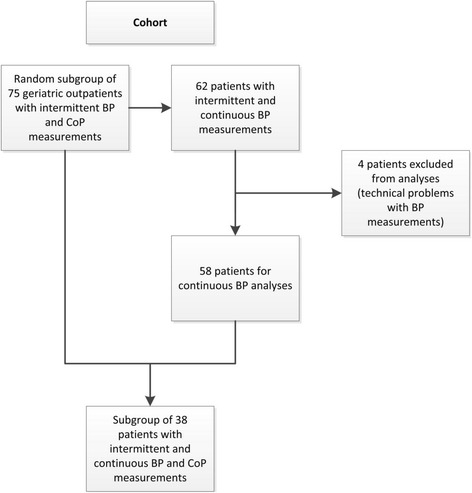
Flowchart of patients used for analyses

This cross-sectional study included a random subgroup of 75 patients measured within an inception cohort of geriatric outpatients who were consecutively referred to the outpatient clinic of a middle-sized teaching hospital (Bronovo hospital, The Hague, The Netherlands) between March 2011 and January 2012. This subgroup is part of a bigger cohort, which was described in detail earlier [20]. Patients were referred to the outpatient clinic by a general practitioner for reasons including but not limited to, mobility problems, falls, complaints of dizziness and/or memory problems. All patients underwent a comprehensive geriatric assessment (CGA) assessing somatic, psychological and social factors, obtained during a two-hour visit. Both intermittent BP measurements and CoP measurements were available in 75 patients. Due to availability of equipment (continuous BP measurements after June 2011) continuous blood pressure data was available in 62 patients. Data of four patients were excluded because of technical problems, leaving 58 patients for analysis. In 38 patients of this subgroup, complete data on intermittent BP, CoP measurements and continuous BP measurements were available. See Fig. 1 for a visual representation. The institutional review board of the Leiden University Medical Center (Committee Medical Ethics (CME), Leiden, the Netherlands) reviewed and approved the study. The need for individual informed consent was waived, as this retrospective research was based on regular clinical care.

### Characteristics of geriatric outpatients

Questionnaires were used to obtain information about age, sex, living situation, current smoking, history of falls during the preceding twelve months, self-reported impaired standing balance and use of walking aid. Body mass index (BMI) was calculated using measurements of height and weight, using a bioelectrical impedance analysis or a scale if patients had a pacemaker, to the nearest decimal (0.1). The number of medication used and information on diseases was extracted from medical charts. Multimorbidity was defined as the presence of two or more diseases, including chronic obstructive pulmonary disease, diabetes mellitus, hypertension, malignancy, myocardial infarction, Parkinson’s disease and (osteo)arthritis. The Mini Mental State Examination (MMSE) was used to assess global cognitive functioning [24]. Handgrip strength was measured in standing position with arm stretched and parallel to the body using a hand dynamometer (Jamar, Sammons Preston, Inc., Bolingbrook, IL, USA). Three trials were performed alternately for each hand; maximum performance of both hands was determined [25]. Gait speed was measured with a 10 m walking test during preferred pace. The Short Physical Performance Battery (SPPB) was used to assess physical functioning. The SPPB includes the ability to maintain standing balance with eyes open in three different standing positions, a timed chair stand test and a timed four meter walking test [26].

### Measurement protocol and BP measurements

Postural changes were imposed after lying supine and fully supported for at least 5 min by an automatic lift chair (Vario 570, Fitform B.V., Best, The Netherlands) assisting the patients to a full standing position with eyes open in a standardized way, i.e. controlling the speed of transition from supine to standing position. Patients subsequently resumed full standing position in an active way.

Intermittent BP measurements were performed using an automated sphygmomanometer on the left arm (Welch Allyn, Skaneateles, USA). Supine BP was measured preceding postural change and after the patient spent at least 5 min in supine position. After 1 and 3 min in standing position, BP measurements were repeated. BP change was determined by subtracting BP at 1 or 3 min from the supine BP; a positive BP change therefore indicates a decrease in BP from supine to standing position.

In the random subgroup (*n* = 38), BP was measured continuously and non-invasively concurrent with the intermittent BP measurement during supine and standing position using a digital photoplethysmograph with a cuff placed on the right middle finger (Finometer PRO, Finapres Medical Systems BV, Amsterdam, The Netherlands) [27]. Beat-to-beat BP data was obtained using BeatScope 1.1 software (Finapres Medical systems BV, Amsterdam, The Netherlands). Beat-to-beat BP data was averaged over 5 s time periods using MATLAB (The MathWorks, Natick, Mass., USA) [28]. Supine BP was defined as the mean BP during the last 60 s in supine position preceding postural change. During the 3 min of stance, twelve consecutive time periods of 15 s were examined. BP change was calculated by subtracting the lowest BP per interval from the supine BP for each time period, yielding 12 measurements.

OH was defined as a drop in systolic BP (SBP) of at least 20 mmHg and/or a drop in diastolic BP (DBP) of 10 mmHg or more within 3 min of standing position [15]. OH _intermittent_ was defined using the BP change at 1 min and 3 min of standing position. Presence of OH_continuous_ was determined for every consecutive time period of 15 s, during the 3 min of standing position. iOH was defined as a transient decrease in BP within 15 s after postural change with a decline in SBP of at least 40 mmHg and/or 20 mmHg in DBP [16] and could only obtained from the continuous BP measurements.

### Center of pressure movement

CoP movement was measured directly after standing up, concurrently with BP measurements, during the 3 min of standing position on a triangular 6 degrees of freedom force plate (ForceLink BV, Culemborg, The Netherlands). A trigger was sent to the force plate by the experimenter at the moment the patient was standing on the force plate. As a safety measure, a support was present in case the patient needed some assistance to prevent from actual falling. Data were recorded with a sample frequency of 1 kHz and were processed in MATLAB (The MathWorks, Natick, Mass., USA). Before analysis, data were low-pass filtered with a cut-off frequency of 10 Hz. CoP movement was expressed in five different CoP parameters (i.e. mean amplitude, amplitude variability, range, mean velocity and velocity variability) and were calculated per 15 s time period for the entire duration of standing upright. For each CoP parameter the time period of its maximal value representing maximum CoP movement (maximum CoP) was determined [29]. Each CoP parameter was transformed into standardized CoP parameters, resulting in Z-score. Direction-specific CoP composite scores (i.e. anterior-posterior (AP) and medial-lateral (ML) direction) were calculated from the standardized single CoP parameters for each consecutive time period by averaging Z-scores of the CoP parameters [22]. Both CoP composite scores and single CoP parameters in AP and ML direction were used for further analysis.

### Statistical analyses

Mean and standard deviation (SD) are used to present continuous variables with a Gaussian distribution. Continuous variables with a non-Gaussian distribution are presented as median and interquartile range (IQR).

**Fig. 2:**
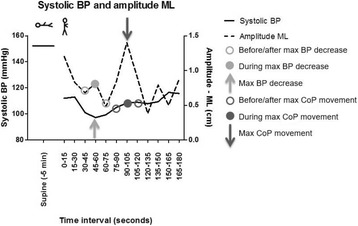
Systolic blood pressure (BP) change (black line) and Center of Pressure (CoP) amplitude in medial-lateral (ML) direction (dotted line) of a representative patient during supine position and over 3 min after postural change. The light grey arrow represents the moment of maximum BP change, while the light grey closed circle and open circles represent respectively the CoP during and the CoP before and after maximum BP change. The dark grey arrow represents the moment of maximum CoP movement, while the dark grey closed circle and open circles represent respectively the BP change during and the BP change before and after maximum CoP movement

Spearman’s rho correlation analysis was used to assess the association between BP change and CoP movement in three ways: (i) the correlation between intermittently measured BP change at 1 and 3 min and CoP parameters respectively in the 15 s intervals before (45-60 s) and after (60-75 s) 1 min of standing, and in the 15 s interval before (165-180 s) 3 min of standing; (ii) the correlation between the continuously measured maximum BP change and the CoP parameters in the 15 s intervals before, during and after the maximum BP change; (iii) the correlation between the maximum of each CoP parameter and the BP change in the 15 s intervals before, during and after the maximum CoP. As only SBP showed the largest change, this parameter was used for further analysis. Figure 2 shows a visual representation of the abovementioned analysis, with the SBP change and CoP amplitude in ML-direction of a representative patient during supine position and over 3 min after postural change. To minimize type I errors, a Bonferroni correction was applied and the alpha was set at 0.005.

Mann-Whitney *U*-test was used to assess possible differences in each single CoP parameter between the OH_continuous_ group and non-OH_continuous_ group. Each CoP parameter was averaged between 30 and 180 s after standing, since it was previously shown that patients needed at least 30 s to find their balance after postural change, regardless of having OH or not [30]. For the Mann-Whitney *U* test, the median of each single CoP parameter was determined and compared between the OH and non-OH group. *P* values lower than 0.05 were considered statistically significant for the Mann-Whitney U-test.

Both iOH and OH_intermittent_ groups were too small to use in separate analyses.

Statistical Package for the Social Sciences (SPSS Inc., Chicago, USA) version 20.0 was used for statistical analyses. GraphPad Prism version 5.01 was used to perform visualization.

## Results

### Characteristics of geriatric outpatients

**Fig. 3:**
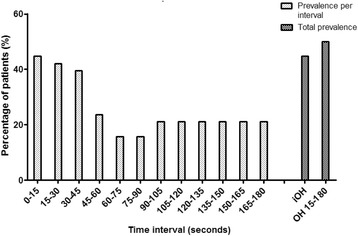
Prevalence of patients with OH_continuous_ per 15 s time period, iOH and total OH_continuous_ in subgroup (*n* = 38) where blood pressure was measured continuously. OH = orthostatic hypotension. iOH = initial orthostatic hypotension

Table 1 shows the characteristics of the geriatric outpatients. Mean age was 80.4 (SD 7.4) years and 33 (44%) patients were male. 46 (61.3%) patients reported a fall incident in the previous 12 months and 32 (43.3%) patients reported impaired standing balance. OH_intermittent_ was found in 8 (11%) patients. In the subgroup in which continuous BP data was available, OH_continuous_ was present in 22 out of 38 patients (57.9%). Three (13.6%) patients had only iOH. Figure 3 presents the prevalence of patients with OH_continuous_ for each time period.

**Table 1:** Characteristics of geriatric outpatients (*n* = 75) and a subgroup of outpatients who underwent continuous blood pressure measurements (*n* = 38)

	All *n* = 75	Subgroup *n* = 38
Socio-demographics		
Age, years	80.4 (7.4)	79.3 (7.7)
Males; *n *(%)	33 (44.0)	19 (50.0)
Independent living; *n *(%)	42 (56.0)	18 (47.4)
Current smoking; *n *(%)	14 (18.7)	6 (15.8)
Health characteristics		
BMI, kg/m^2^	25.8 (5.2)	26.5 (5.6)
Hypertension; n (%)	29 (39.7)	16 (42.1)
Diabetes Mellitus; *n *(%)	20 (27.4)	11 (28.9)
Parkinson’s disease; *n *(%)	1 (1.4)	0 (0)
Multimorbidity, *n *(%)^a^	28 (38.4)	16 (42.1)
Number of medication; median [IQR]	5 [2-8]	5 [3-7]
MMSE, points; median [IQR]	28 [25-29]	28 [25-29]
Physical functioning		
Handgrip strength, kg	27.1 (7.3)	28.4 (7.3)
Gait speed, m/s	0.81 (0.31)	0.78 (0.31)
SPPB, points; median [IQR]	8 [6-10]	8 [6-11]
Self-reported		
Fall incident previous 12 months; *n *(%)	46 (61.3)	24 (63.2)
Impaired standing balance; *n *(%)	32 (43.3)	13 (35.1)
Use of walking aid; *n* (%)	41 (55.4)	20 (52.6)
Supine blood pressure ^b^		
SBP, mmHg	135 (22.1)	140 (23.0)
DBP, mmHg	73 (9.8)	75 (10.2)
Blood pressure change after postural change		
SBP change, mmHg ^c^		
After 1 min	0.64 (13.89)	−1.51 (14.9)
After 3 min	−3.97 (14.5) ^f^	−4.40 (15.7)
DBP change, mmHg ^c^		
After 1 min	−4.36 (7.21)	−3.46 (7.18)
After 3 min	−6.07 (8.98) ^f^	−6.14 (10.6)
Orthostatic hypotension ^d^		
OH_intermittent_; *n* (%)	8 (11.0)	3 (8.1)
OH_continuous_0-180_; *n* (%)	NA	22 (57.9)
iOH ^e^ and OH 15-180 s	NA	14 (63.6) ^g^
Only iOH ^e^	NA	3 (13.6) ^g^

All parameters are presented as mean (standard deviation) unless indicated otherwise

*BMI* Body Mass Index, *IQR* interquartile range, *MMSE* Mini Mental State Examination, *SPPB* Short Physical Performance Battery, *BP* blood pressure, *SBP* systolic blood pressure, *DBP* diastolic blood pressure, *OH* orthostatic hypotension, *iOH* Initial orthostatic hypotension, *NA* not applicable

^a^Two or more chronic diseases, including chronic obstructive pulmonary diseases, diabetes mellitus, hypertension, malignancy, myocardial infarction, Parkinson’s disease, (osteo)arthritis

^b^Measured after at least 5 min in supine position

^c^Supine BP minus BP at 1 or 3 min after postural change, intermittently measured

^d^A decrease in SBP of ≥20 mmHg or decrease in DBP of ≥10 mmHg at 1 or at 3 min after postural change, intermittently measured

^e^Transient decrease in BP within 15 s after standing, a > 40 mmHg decrease in SBP and/or a > 20 mmHg decrease in DBP

^f^Data available of *N* = 69

^g^From patients with OH_continuous_0-180_, (*N* = 22)

From the patients with OH 5 out of 22 (22.7%) reported complaints during postural change. No black out or light headedness was reported. Two patients (9.1%) reported being dizzy, while 3 patients (13.6%) reported being unstable.

### Association between BP change and CoP movement

#### Intermittent BP measurement

No significant associations were found between intermittently measured BP change and CoP parameters. The range of r was between −0.20 and 0.31 for the association between SBP and AP CoP parameters, with a minimal *p*-value of 0.01. For the association between SPB and ML CoP parameters the range of r was between −0.15 and 0.16 with a minimal *p*-value of 0.21.

The association between DBP and AP CoP parameters had a range of r between −0.20 and −0.04 with a minimal *p*-value of 0.09. The association between DBP and ML CoP parameters had a range of r between −0.08 and 0.13 with a minimal *p*-value of 0.29.

#### Continuous BP measurement

No association was found between the continuously measured maximum BP change and the CoP parameters 15 s before, during and after the maximum BP change. Range of r was between −0.26 and 0.20 for AP CoP parameters with a minimal *p*-value of 0.26, and between −0.52 and 0.20 for ML CoP parameters with a minimal *p*-value of 0.13 (Additional file 1: Table S1).

Table 2 shows the association between the maximum of each CoP parameter and continuously measured BP change 15 s before, during and after the maximum CoP.

**Table 2:** Association between maximum Center of Pressure (CoP) movement for both AP and ML direction and continuously measured BP change 15 s before (*n* = 25), during (*n* = 38) and after (*n* = 36) after the maximum CoP movement

		BP change					
		Before^a^		During^a^		After^a^	
		*r*	*p*-value	*r*	*p*-value	*r*	*p*-value
AP	Mean amplitude (cm)	.23	.31	.14	.40	.29	.10
	Amplitude variability (cm)	−.05	.82	.04	.81	.09	.63
	Range (cm)	−.19	.39	.10	.55	.04	.82
	Mean velocity (cm/s)	−.12	.65	−.05	.76	−.01	.95
	Velocity variability (cm/s)	.08	.72	.00	.98	.02	.92
ML	Mean amplitude (cm)	.36	.09	.33	.04	.11	.56
	Amplitude variability (cm)	.07	.76	.16	.36	.10	.58
	Range (cm)	−.13	.54	.10	.56	.00	.99
	Mean velocity (cm/s)	−.28	.25	.09	.60	.17	.33
	Velocity variability (cm/s)	−.28	.25	.14	.39	.15	.40

*BP* systolic blood pressure, *CoP* center of pressure, *AP* anterior-posterior, *ML* medial-lateral

*p*-values obtained with Spearman’s rho correlation analysis. Bonferroni adjusted *p*-value of .005 was statistically significant. Analyses were done in 15 s time periods

^a^Maximum CoP movement

No significant associations were found between the maximum BP change and CoP parameters and between the maximum CoP and BP change.

### Differences in CoP movement between patients with and without OH_continuous_

Table 3 shows the median and IQR of each CoP parameter averaged between 30 and 180 s in both AP and ML direction of the OH_continuous_ group and the non-OH_continuous_ group. No significant differences were found for the median of CoP parameters comparing the OH_continuous_ and non- OH_continuous_ group.

**Table 3:** Center of Pressure (CoP) parameters in both anterior-posterior (AP) and medial-lateral (ML) direction averaged between 30 and 180 s for both the group of patients with OH_continuous_ (*n* = 22) and non-OH_continuous_ (*n* = 16)

		OH_continuous_	Non-OH_continuous_	
		Median [IQR]	Median [IQR]	*p*-value
AP	Mean amplitude (cm)	0.56 [0.31 – 0.82]	0.41 [0.31 – 0.52]	.60
	Amplitude variability (cm)	0.35 [0.25 – 0.54]	0.31 [0.23 – 0.46]	.39
	Range (cm)	1.85 [1.28 – 2.70]	1.60 [1.07 – 2.29]	.36
	Mean velocity (cm/s)	4.87 [4.13 – 5.26]	4.56 [4.17 – 5.58]	.60
	Velocity variability (cm/s)	7.20 [6.17 – 7.91]	6.86 [6.11 – 8.34]	.60
ML	Mean amplitude (cm)	0.55 [0.43 – 0.77]	0.55 [0.42 – 0.61]	.44
	Amplitude variability (cm)	0.54 [0.40 – 0.77]	0.47 [0.37 – 0.62]	.29
	Range(cm)	2.66 [1.98 – 3.69]	2.18 [1.87 – 2.97]	.29
	Mean velocity (cm/s)	3.64 [3.05 – 4.19]	3.39 [2.81 – 3.70]	.12
	Velocity variability (cm/s)	5.13 [4.59 – 5.91]	4.95 [3.94 – 5.20]	.10

All parameters are given as median [IQR]. *p*-values obtained with Mann-Whitney-U test

*OH*_*continuous*_ orthostatic hypotension; continuously measured, *IQR* interquartile range, *AP* anterior-posterior, *ML* medial-lateral

## Discussion

This study aimed to assess the association between BP change after postural change and quality of standing balance, in a clinically relevant population of geriatric outpatients, using both intermittently and continuously measured BP. No significant associations were found between BP change, both measured intermittently and continuously, and CoP movement. Furthermore, no significant differences were found in CoP movement between patients with or without continuously measured OH. Thus, no relation between BP and CoP could be established.

The absence of associations was in contrast to our hypotheses and most literature [3, 11, 20, 21]. The relationship between physical functioning, falls and orthostatic hypotension in the same cohort of geriatric outpatients was assessed previously and showed that blood pressure decrease after postural change was associated with increased self-reported impaired standing balance and falls and with a reduced ability to maintain standing balance in semi-tandem stance [20]. Previous studies showed that BP change or OH, measured intermittently or continuously, was found to be associated with either increased postural sway, measured as CoM, or impaired standing balance, using clinical balance tests [3, 11, 20, 21]. Differences may be explained by a variety of factors.

First, we investigated a clinically relevant population of geriatric outpatients whereas the study populations in the aforementioned studies consisted of community-dwelling healthy older adults [3], older patients with hypertension from a geriatric ward [11] and older adults with idiopathic Parkinson’s disease [21]. The heterogeneity of the study population as a subsample of patients referred to an outpatient clinic of a teaching hospital, may have caused attenuation of effects of BP changes in their relation to CoP by risk factors as hypertension and use of medication. Although, up to 39.7% in all 75 and 42.1% in the subgroup of 38 patients had hypertension, still effects are unclear (see also Additional file 2: Table S2).

Second, conflicting results can be explained by the use of a different measure of BP. In this study, we use continuous BP measurements, which give a more actual representation of BP change. Only in our previous study BP was measured continuously [20], while the other aforementioned studies have measured BP intermittently [3, 11, 21]. Furthermore, we used a different measure of standing balance, which may also explain the conflicting results. CoP movement portrays the quality of standing balance and can be used as a measure for impaired standing balance [22, 23]. However, CoP movement is an indirect measure of standing balance [31]. In the present study, CoP movement was used as a measure of quality of standing balance, while other studies used CoM movement, as a measure of postural sway by an inclinometric instrument [21], an ataxiameter [3] or the ability to maintain balance during side-by-side and tandem stance [11, 20]. Furthermore, in all, except for one study [11] standing balance was not measured directly after standing up. Measuring standing balance directly after standing up gives the most actual representation of standing balance in daily life. Not measuring standing balance directly after standing up may give results differing from an ecological situation. Furthermore, it does not take iOH into account, which is significantly associated with impaired standing balance in older adults and therefore plays an important role in falling [4, 5].

Next to differences in study set up, the absence of association may also be explained by the underlying pathophysiological mechanisms, especially the understanding of compensatory mechanisms as cerebral autoregulation, which are still unresolved [32–35]. Cerebral autoregulation modulates cerebral blood flow and perfusion and is influenced by impaired BP regulation [32, 36]. Furthermore, cerebral hypoperfusion might result in reduced neural control. Impaired balance will be the net result of aforementioned mechanisms and may only become apparent when compensatory strategies fail, i.e. cerebral autoregulation and/or balance control. In the present study, cerebral blood flow was not measured directly and CoP movement was used as a measure of standing balance control. Some compensatory strategies were allowed (e.g. comfortable base of support and eyes open standing conditions) to assess standing positions close to an ecological situation.

In order to assess effects of impaired standing balance in daily life in relation to BP change and/or state of OH, it is necessary to assess influence of compensatory strategies; full assessment comprising CoP as well as CoM movement may be required as well as measurements under daily life conditions. In the present study, patients were standing with eyes open on the force plate, thus enabling the body to use visual input for balance control. Previous studies found impaired standing balance, independent of BP, to be present in eyes-closed conditions [20, 22, 37, 38] and thereby eliminating compensatory mechanisms as sensory reweighting [20]. Moreover, patients in the present study stood in their preferential stance to mimic ecological conditions during daily life, where standing with a wider base of support might be another compensation mechanism to overcome decreased quality of standing balance. Disentanglement of cause- and effect interrelations may require sophisticated methods encompassing external perturbations [7, 22].

### Strengths and limitations

Data was derived from a clinically relevant population of geriatric outpatients. Continuous BP measurement was used concurrently with CoP measurements, while patients stood in a way closely resembling an ecological situation. A limitation of this study is the cross-sectional design, which makes it impossible to draw any conclusive information about a causal relationship between BP change and standing balance. Only the number of medications was recorded so that influence of blood pressure regulating medication could not be addressed. Another limitation is the small sample size used for the analyses preventing adjustment of covariates as medication and hypertension. With the currently used methods it is difficult to distinguish the underlying systems because of mutual interaction. Especially it is of importance to address the role of compensatory mechanisms as cerebral autoregulation [7].

## Conclusions

Larger BP change and having OH or not did not relate to altered CoP movement after postural change in a relevant group of geriatric outpatients. Future research should focus on using continuously measured BP and cerebral perfusion measurements in all clinically relevant groups of patients and different measure of standing balance, i.e. between CoP movement and CoM movement to advance our understanding on the effect of OH on standing balance. Moreover, research should focus on a better understanding of the pathophysiological mechanisms of impaired balance and OH.

## Additional files

Additional file 1: Table S1. Association between continuously measured maximum BP change and Center of Pressure (CoP) movement 15 s before (*n* = 10), during (*n* = 35) and after (*n* = 36) maximum BP change. (DOCX 16 kb)

Additional file 2: Table S2. Association between intermittently measured BP change at 1 min and Center of Pressure (CoP) movement in the period 45-60s (15 s before BP measurement, *n* = 72) and 60-75 s (15 s after BP measurement, *n* = 72) and between intermittently measured BP change at 3 min and CoP movement in the period 165-180 s (15 s after, *n* = 59). (DOCX 18 kb)

## Abbreviations

AP: Anterior-posterior
BMI: Body mass index
BP: Blood pressure
CoM: Center of mass
CoP: Center of pressure
DBP: diastolic blood pressure
iOH: initial orthostatic hypotension
ML: Medial-lateral
MMSE: Mini Mental State Examination
OH: Orthostatic hypotension
SBP: Systolic blood pressure
SPPB: Short Physical Performance Battery
