# Supplementary material for: Blood pressure change does not associate with Center of Pressure movement after postural transition in geriatric outpatients
Source: BMC Geriatr. 2018 Jan 15;18:10. doi: 10.1186/s12877-017-0702-2 (PMC5769426; doi:10.1186/s12877-017-0702-2)
Supplement: Supplementary file 1 — Association between continuously measured maximum BP change and Center of Pressure (CoP) movement 15 s before (n = 10), during (n = 35) and after (n = 36) maximum BP change. (DOCX 16 kb) [file 12877_2017_702_MOESM1_ESM.docx]

**Additional file 1: Table S1**. Association between continuously measured maximum BP change and Center of Pressure (CoP) movement 15 seconds before (n=10), during (n=35) and after (n=36) maximum BP change

|  |  | Maximum BP change | | | | | | | |
| --- | --- | --- | --- | --- | --- | --- | --- | --- | --- |
|  |  | Before | |  | During | |  | After | |
|  |  | r | p-value |  | r | p-value |  | r | p-value |
| AP | Composite score | -.19 | .57 |  | .02 | .91 |  | .04 | .84 |
|  | Mean amplitude (cm) | -.24 | .51 |  | .11 | .54 |  | .00 | .99 |
|  | Amplitude variability (cm) | -.26 | .47 |  | .03 | .87 |  | .11 | .51 |
|  | Range (cm) | -.18 | .59 |  | .05 | .77 |  | .20 | .56 |
|  | Mean velocity (cm/s) | -.18 | .63 |  | .18 | .30 |  | .14 | .42 |
|  | Velocity variability (cm/s) | -.15 | .68 |  | .20 | .26 |  | .18 | .30 |
| ML | Composite score | -.05 | .89 |  | .10 | .58 |  | .18 | .29 |
|  | Mean amplitude (cm) | -.52 | .13 |  | .00 | .98 |  | .01 | .96 |
|  | Amplitude variability (cm) | .03 | .93 |  | .06 | .72 |  | .14 | .43 |
|  | Range (cm) | .18 | .59 |  | .02 | .91 |  | .14 | .41 |
|  | Mean velocity (cm/s) | .02 | .96 |  | .16 | .35 |  | .16 | .36 |
|  | Velocity variability (cm/s) | .03 | .93 |  | .20 | .25 |  | .19 | .26 |

BP: blood pressure, CoP: center of pressure, AP: anterior-posterior, ML: medial-lateral. p-values obtained with Spearman’s rho correlation analysis. Bonferroni adjusted alpha of .005 was considered statistically significant.
