# Supplementary material for: Blood pressure change does not associate with Center of Pressure movement after postural transition in geriatric outpatients
Source: BMC Geriatr. 2018 Jan 15;18:10. doi: 10.1186/s12877-017-0702-2 (PMC5769426; doi:10.1186/s12877-017-0702-2)
Supplement: Supplementary file 2 — Association between intermittently measured BP change at 1 min and Center of Pressure (CoP) movement in the period 45-60s (15 s before BP measurement, n = 72) and 60-75 s (15 s after BP measurement, n = 72) and between intermittently measured BP change at 3 min and CoP movement in the period 165-180 s (15 s after, n = 59). (DOCX 18 kb) [file 12877_2017_702_MOESM2_ESM.docx]

Additional file 2 **Table S2**. Association between intermittently measured BP change at 1 minute and Center of Pressure (CoP) movement in the period 45-60s (15 s before BP measurement, n=72) and 60-75s (15 s after BP measurement, n=72) and between intermittently measured BP change at 3 minutes and CoP movement in the period 165-180s (15 s after, n=59)

|  |  | 45-60s | | | | |  | 60-75s | | | | |  | 165-180s | | | | |
| --- | --- | --- | --- | --- | --- | --- | --- | --- | --- | --- | --- | --- | --- | --- | --- | --- | --- | --- |
|  |  | SBP | | DBP | | |  | SBP | | DBP | | |  | SBP | | DBP | | |
|  |  | r | p-value |  | r | p-value |  | r | p-value |  | r | p-value |  | r | p-value |  | r | p-value |
| AP | Composite score | .11 | .36 |  | -.11 | .34 |  | .10 | .40 |  | -.16 | .17 |  | -.04 | .78 |  | -.15 | .25 |
|  | Mean amplitude (cm) | .03 | .82 |  | -.12 | .32 |  | -.14 | .24 |  | -.20 | .09 |  | -.15 | .26 |  | -.19 | .14 |
|  | Amplitude variability (cm) | -.20 | .10 |  | -.08 | .53 |  | -.10 | .42 |  | -.08 | .53 |  | .01 | .97 |  | -.11 | .42 |
|  | Range (cm) | -.19 | .11 |  | -.04 | .72 |  | -.17 | .16 |  | -.12 | .34 |  | .04 | .78 |  | -.07 | .59 |
|  | Mean velocity (cm/s) | .22 | .06 |  | -.14 | .24 |  | .29 | .01 |  | -.10 | .41 |  | -.02 | .91 |  | -.10 | .44 |
|  | Velocity variability (cm/s) | .25 | .04 |  | -.14 | .25 |  | .31 | .01 |  | -.10 | .42 |  | -.02 | .89 |  | -.10 | .45 |
| ML | Composite score | -.08 | .51 |  | .04 | .74 |  | .03 | .78 |  | .04 | .75 |  | .12 | .37 |  | .09 | .51 |
|  | Mean amplitude (cm) | -.13 | .30 |  | -.05 | .69 |  | .05 | .67 |  | .04 | .73 |  | .03 | .83 |  | .04 | .76 |
|  | Amplitude variability (cm) | -.15 | .21 |  | .10 | .41 |  | -.08 | .50 |  | .05 | .65 |  | .13 | .31 |  | .07 | .59 |
|  | Range (cm) | -.14 | .23 |  | .13 | .29 |  | -.09 | .45 |  | .06 | .63 |  | .16 | .23 |  | .12 | .36 |

**Table 1**. (*continued*)

|  |  | 45-60s | | | | |  | 60-75s | | | | |  | 165-180s | | | | |
| --- | --- | --- | --- | --- | --- | --- | --- | --- | --- | --- | --- | --- | --- | --- | --- | --- | --- | --- |
|  |  | SBP | | DBP | | |  | SBP | | DBP | | |  | SBP | | DBP | | |
|  |  | r | p-value |  | r | p-value |  | r | p-value |  | r | p-value |  | r | p-value |  | r | p-value |
| ML | Mean velocity (cm/s) | .03 | .79 |  | .07 | .54 |  | .09 | .45 |  | -.08 | .52 |  | .08 | .56 |  | .02 | .91 |
|  | Velocity variability (cm/s) | .10 | .39 |  | .10 | .41 |  | .13 | .27 |  | -.07 | .54 |  | .11 | .42 |  | .04 | .76 |

BP: blood pressure, SBP: systolic blood pressure, DBP: diastolic blood pressure, CoP: center of pressure, AP: anterior-posterior, ML: medial-lateral. p-values obtained with Spearman’s rho correlation analysis. Bonferroni adjusted p-value of .005 was statistically significant.
